# Resistin Mediates Sex-Dependent Effects of Perivascular Adipose Tissue on Vascular Function in the Shrsp

**DOI:** 10.1038/s41598-019-43326-z

**Published:** 2019-05-03

**Authors:** Heather Yvonne Small, Sarah McNeilly, Sheon Mary, Adam Marcus Sheikh, Christian Delles

**Affiliations:** BHF Glasgow Cardiovascular Research Centre, Institute of Cardiovascular and Medical Sciences, University of Glasgow, Glasgow, Scotland

**Keywords:** Hypertension, Experimental models of disease

## Abstract

Premenopausal women are relatively protected from developing hypertension compared to men. Perivascular adipose tissue (PVAT) has been shown to mediate vasoactive effects; however, a sex-dependent difference in PVAT function in the setting of hypertension has not yet been explored. We investigated the effect of PVAT on resistance vessel biology in male and female 16 week old stroke prone spontaneously hypertensive rats (SHRSP). This preclinical model of hypertension exhibits a sex-dependent difference in the development of hypertension similar to humans. Wire myography was used to assess vascular function in third-order mesenteric arteries. K_ATP_ channel-mediated vasorelaxation by cromakalim was significantly impaired in vessels from SHRSP males + PVAT relative to females (*maximum relaxation: male* + *PVAT 46.9* ± *3.9% vs. female* + *PVAT 97.3* ± *2.7%*). A cross-over study assessing the function of male PVAT on female vessels confirmed the reduced vasorelaxation response to cromakalim associated with male PVAT (*maximum relaxation: female* + *PVAT*_*female*_ *90.6* ± *1.4% vs. female* + *PVAT*_*male*_ *65.8* ± *3.5%*). In order to explore the sex-dependent differences in PVAT at a molecular level, an adipokine array and subsequent western blot validation identified resistin expression to be increased approximately 2-fold in PVAT from male SHRSP vessels. Further wire myography experiments showed that pre-incubation with resistin (40 ng/ml) significantly impaired the ability of female + PVAT vessels to relax in response to cromakalim (*maximum relaxation: female* + *PVAT 97.3* ± *0.9% vs. female* + *PVAT* + *resistin*_*[40ng/ml]*_ *36.8* ± *2.3%*). These findings indicate a novel role for resistin in mediating sex-dependent vascular function in hypertension through a K_ATP_ channel-mediated mechanism.

## Introduction

Worldwide, hypertension affects more than 40% of people over 25 years of age and is the single biggest risk factor for the development of cardiovascular disease^1^. Overall, there is no difference in the prevalence of hypertension between adult (≥20 years old) men and women. However, it is well-established that men develop hypertension at an earlier age than women^2^. One of the candidates proposed to explain this sex-dependent difference is the presence of estrogen. Estrogen has been shown to be protective against the development of hypertension through acting as a vasodilator, inhibiting the sympathetic nervous system, regulating the renin-angiotensin sytem and by reducing renal damage^3–5^. In spite of this knowledge, the effect of treatments such as hormone replacement therapy have been inconclusive with regard to a beneficial effect of estrogen on the development of cardiovascular disease^6–8^. Research is required to fully delineate the pathophysiological mechanisms underlying the sex-dependent difference in the development of hypertension. Specifically, the role of perivascular adipose tissue (PVAT), an important modulator of vascular function, has yet to be investigated.

PVAT is the fat reserve surrounding blood vessels and consists of adipocytes, immune cells, fibroblasts and endothelial cells^9,10^. Depending on its location, it has the characteristics of either brown or white adipose tissue. The former is thermogenic and primarily involved in energy expenditure, whereas the latter is believed to serve as energy storage. The thoracic aorta is surrounded by brown adipose tissue, whereas the abdominal aorta and mesenteric arteries are surrounded by white adipose tissue^9^. In addition to acting as structural support and protection for vessels, PVAT also has direct effects on the vasculature^11^. This phenomenon was first discovered in 1991 by Soltis and Cassis who showed that PVAT asserted anti-contractile effects on vessels through so-called ‘adventitium derived relaxing factors’ (ADRF)^10^. These ADRFs affect vasorelaxation by acting through the opening of potassium channels leading to calcium influx and hyperpolarisation. These mechanisms can be both endothelium-dependent and independent. Several ADRF candidates have been proposed experimentally, such as, adiponectin, angiotensin 1–7, hydrogen sulphide and prostacyclins^12^. With respect to hypertension, studies in a number of preclinical models have concluded that a reduction of PVAT mediated anti-contractile effect contributes to an increase in blood pressure^13–16^. Underlying causes have been attributed to the increased release of inflammatory factors stimulating the production of contractile agents such as angiotensin II and superoxide from the PVAT. Further, a reduction in ADRF or potassium channels in the PVAT or vascular smooth muscles cells may also contribute^17^.

Here, we hypothesize that there is an alteration in PVAT function between males and females, which contributes to the sex-dependent difference observed in the development of hypertension. We utilized the stroke-prone spontaneously hypertensive rat (SHRSP) to investigate the differences between male and female PVAT. The SHRSP is a well-established model of cardiovascular disease sharing many of the pathologies seen in humans; including the sex difference observed in hypertension^18^. This difference is observed from 12 weeks of age, but more pronounced at 16 weeks, when the systolic blood pressure of male SHRSP is approximately 30 mmHg higher than female SHRSP^19^. This study used age-matched 16 week old male and female SHRSP to explore sex-dependent differences in PVAT function in hypertension using *ex vivo* and molecular-based studies.

## Methods

### Animals

Animals (strain: SHRSP and WKY) were housed under controlled lighting (0700–1900 hours) and temperature (21 ± 3 °C) and received a normal diet (rat and mouse no.1 maintenance diet; Special Diet Services, Grangemouth, United Kingdom) provided *ad libitum*. All animal procedures were approved by the Home Office according to the regulations on experiments with animals in the United Kingdom (Project License Number 60/4286). In this study, all animals were age matched at 16 weeks of age (±4 days). Experiments were carried out according to the guidelines of ARRIVE and Directive 2010/63/EU of the European Parliament on the protection of animals used for scientific purposes. The number of animals used for a given experiment is indicated in the associated figure legend.

### PVAT weight measurement

Prior to the wire-myography experiments, PVAT of male and female WKY and SHRSP was dissected from 3 vessels per animal and weighed to assess the average amount of PVAT per vessel.

### Wire myography

Rings for wire myography were consistently prepared from third-order mesenteric arteries (average diameter 250 µm) harvested in physiological salt solution (PSS) (0.25 M NaCl, 0.001 M KCl, 2 mM MgSO_4_, 50 mM NaHCO_3_, 2 mM KH_2_PO_4_, 1 mM glucose, 2.5 mM CaCl_2_) from 16 week old male and female WKY and SHRSP. Mesenteric artery rings (1.8–2.0 mm in length) with or without (+/−) PVAT were mounted on two stainless steel wires on a four channel small vessel myograph (AD Instruments, Oxford, UK) in PSS. Vessels were normalized and subject to a wake-up procedure using high potassium PSS^1^. To establish the vessel’s contractile response, noradrenaline (Sigma-Aldrich, Dorset, UK) was added at the following increasing concentrations: 1 × 10^−9^, 1 × 10^−8^, 1 × 10^−7^, 1 × 10^−6^, 3 × 10^−6^, 1 × 10^−5^ and 3 × 10^−5^ M. To determine the vessel’s endothelium-dependent relaxation response, vessels were pre-constricted with 1 × 10^−5^ M noradrenaline followed by the addition of carbachol (Sigma-Aldrich, Dorset, UK) at the following increasing concentrations: 1 × 10^−9^, 1 × 10^−8^, 1 × 10^−7^, 1 × 10^−6^ and 1 × 10^−5^ M. To assess K_ATP_ channel mediated relaxation, vessels were pre-constricted with 1 × 10^−5^ M noradrenaline followed by the addition of cromakalim (Sigma-Aldrich, Dorset, UK) at following concentrations: 1 × 10^−9^, 1 × 10^−8^, 1 × 10^−7^, 1 × 10^−6^, 1 × 10^−5^ and 3 × 10^−5^ M. At the low concentration used, cromakalim could be dissolved in PSS. SNP was used as a positive control for vasorelaxation.

### Resistin pre-incubation

For wire myography experiments carried out to assess the effect of resistin, vessels were mounted on the small vessel wire myograph followed by incubation with 40 ng/ml recombinant rat resistin (Sigma-Aldrich, Dorset, UK) for 4 hours at 37 °C. Following incubation, vessels were subject to dose-response curves as described in “Wire myography”.

### Cross-over study

To determine the sex specific effects of male and female PVAT, a cross-over study was devised. For this, PVAT was dissected from female vessels, weighed (average weight approximately 10 µg) and attached to the heads of the small vessel myograph with a stainless steel wire (Fig. S1). PVAT was used on the same day as it was harvested in these studies. This ensured close proximity of the vessel to the PVAT. Attaching the PVAT best replicates the physiologically relevant setting of endogenous PVAT. Prior to the experiment, the set up was compared to vessels with intact PVAT to exclude potential interference of the additional wire (Fig. S4). The same procedure was carried out using male PVAT on female vessels. Vessels were subject to dose-response curves as described in “Wire myography”.

### Histopathology

Mesenteric vessels with intact PVAT were harvested from male and female WKY and SHRSP and fixed for 24 hours in 10% formalin. Paraffin sections of 5 µm were used for staining. Immediately prior to staining, slides were deparaffinised and rehydrated through an ethanol gradient into distilled H_2_O. Sections were stained with haematoxylin and eosin using standard protocol. This was followed by 1 minute under running tap water and dehydration through an ethanol gradient and mounted using DPX (Sigma-Aldrich, Dorset, UK). Sections were viewed using light microscopy taken at 20x objective. Adipocyte density and size was quantified using Image J (National Institutes of Health, Bethesda, USA). Sections were analysed by an operator who was blinded to the identity of the slides.

### Adipokine array

The rat Proteome Profiler^™^ array (R&D Systems, Abingdon, UK) was carried out according to manufacturer’s instructions. In brief, snap frozen tissue was homogenized in PBS with protease inhibitors (Roche Laboratories, Welwyn Garden City, UK) and frozen in 1% Triton-X at −80 °C before use. Total protein concentration was then assessed using Bradford reagent (Sigma-Aldrich, Dorset, UK) where 250 µg was used for the adipokine array. Biotinylated antibodies were then mixed with the sample and incubated with the membrane overnight at 4 °C. The next day, the membrane was washed and incubated with Strepdavidin-HRP. After further washes, Chemi Reagent mix was distributed evenly on the membranes and visualised using the Licor Ci-DiGit (Licor, Cambridge, UK).

### Western blot

Snap frozen tissue was homogenized in PBS with protease inhibitors (Roche Laboratories, Welwyn Garden City, UK) and frozen in 1% Triton-X at −80 °C before use. Protein concentration was measured using the Pierce™ BCA Protein Assay Kit (Thermo Fisher, Renfrew, UK). A total of 30 µg was loaded into each well. The membrane was blocked with 5% fat-free milk before incubation with the primary antibody for resistin (#ab93069, Abcam, Cambridge, UK). Membranes were incubated with an anti-chicken HRP-conjugated secondary antibody (#ab191865, Abcam, Cambridge, UK). Proteins were detected using Amersham enhanced chemiluminescence (ECL) western blotting detection reagents (GE Life Sciences, Buckinghamshire) in a 1:1 ratio and visualised with the Licor Ci-DiGit (Licor, Cambridge, UK).

### Data analysis

Statistical analysis was performed using performed using GraphPad Prism version 4.0 for Windows (GraphPad Software, La Jolla California, USA). Error bars in all graphs are indicative of standard error of the mean. A Student’s t-test was employed where 2 groups were compared and one way ANOVA followed by post-hoc Tukey test was used to compare >2 groups. The statistical test is given in the associated figure legend. A p-value of < 0.05 was considered to be statistically significant for all experiments.

## Results

### WKY and SHRSP exhibit altered mesenteric artery function in the presence of PVAT

Wire myography was used to assess mesenteric artery function in the presence or absence of PVAT in age-matched (16 week old) male WKY and SHRSP. PVAT had an anti-contractile effect in both WKY and SHRSP; however SHRSP + PVAT vessels were more responsive to noradrenaline than WKY (Fig. S1A,B). K_ATP_ channel-mediated vasorelaxation to cromakalim was not significantly different between WKY and SHRSP or in the presence or absence of PVAT (Fig. S1C). WKY and SHRSP mesenteric arteries +/− PVAT had a similar maximum vasorelaxation response to carbachol and sodium nitroprusside (SNP) (Fig. S1D,E). The presence or absence of PVAT did not significantly alter the response of the vessels to carbachol or cromakalim in either strain (Fig. S1D,E).

When normalised to bodyweight, SHRSP had a greater amount of PVAT surrounding their mesenteric arteries than WKY (Fig. S2A). On histological examination, no strain-dependent difference was observed in adipocyte size or density (Fig. S2B,C).

### Sex-dependent differences exist in PVAT function and structure in the SHRSP

**Figure 1:**
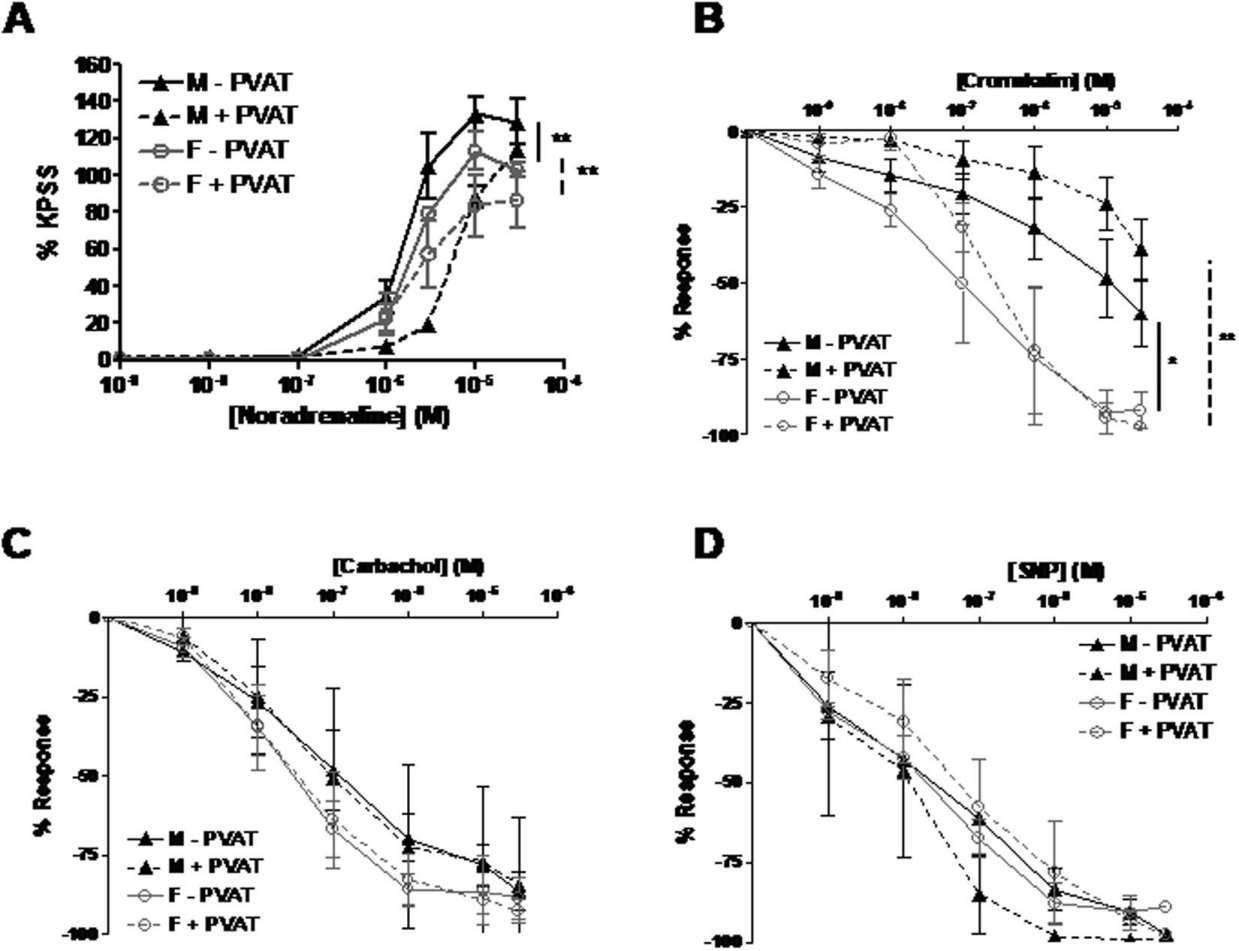
Male and female SHRSP exhibit different third-order mesenteric artery function in the absence and presence of PVAT. Wire myography was used to assess mesenteric artery function in age-matched (16 weeks old) male (n = 5) and female (n = 5) SHRSP. PVAT had an anti-contractile effect in both male and female (**A**). Vessels from male SHRSP - PVAT were more responsive to noradrenaline than vessels from female SHRSP - PVAT (A) (**p < 0.01; EC_50_ SHRSP male 1.84 ± 1.30 μM vs. SHRSP female 3.43 ± 0.41 μM followed by one way ANOVA and post-hoc Tukey test) (**A**). In addition, vessels from male SHRSP + PVAT were more responsive to noradrenaline than vessels from female SHRSP + PVAT (**p < 0.01; EC_50_ SHRSP male 6.13 ± 0.82 μM vs. SHRSP female 12.35 ± 1.27 μM followed by one way ANOVA and post-hoc Tukey test) (**A**). Vasorelaxation to cromakalim was impaired in vessels – PVAT from male SHRSP relative to vessels – PVAT from female SHRSP (*p < 0.05; area under the curve followed by one way ANOVA and post-hoc Tukey test) and in vessels + PVAT from male SHRSP relative to vessels + PVAT from female SHRSP (**p < 0.01; area under the curve followed by one way ANOVA and post-hoc Tukey test) (**B**). There was a trend for endothelium-dependent vasorelaxation to carbachol to be greater in vessels from female relative to male SHRSP; PVAT did not induce a significant difference in carbachol response in either strain (**C**). Vasorelaxation to SNP was not significantly different between strains; PVAT did not significantly alter the response to SNP in either strain (**D**).

Wire myography was used to assess mesenteric artery function in the presence or absence of PVAT in age-matched (16 week old) male and female SHRSP. PVAT had an anti-contractile effect in both male and female SHRSP (Fig. 1A). Mesenteric arteries from male SHRSP had a greater contractile response to noradrenaline than vessels from females both in the presence or absence of PVAT (Fig. 1A). Vasorelaxation to cromakalim was significantly impaired in vessels from male SHRSP relative to female SHRSP (Fig. 1B). Vessels from female SHRSP +/− PVAT exhibited a trend to be more responsive to carbachol than vessels from males; however this was not statistically significant (Fig. 1C). Vasorelaxation to SNP was not significantly different between strains or in the presence or absence of PVAT (Fig. 1D). The equivalent experiment in WKY did not show a sex-dependent difference in vascular function (Fig. S3).

**Figure 2:**
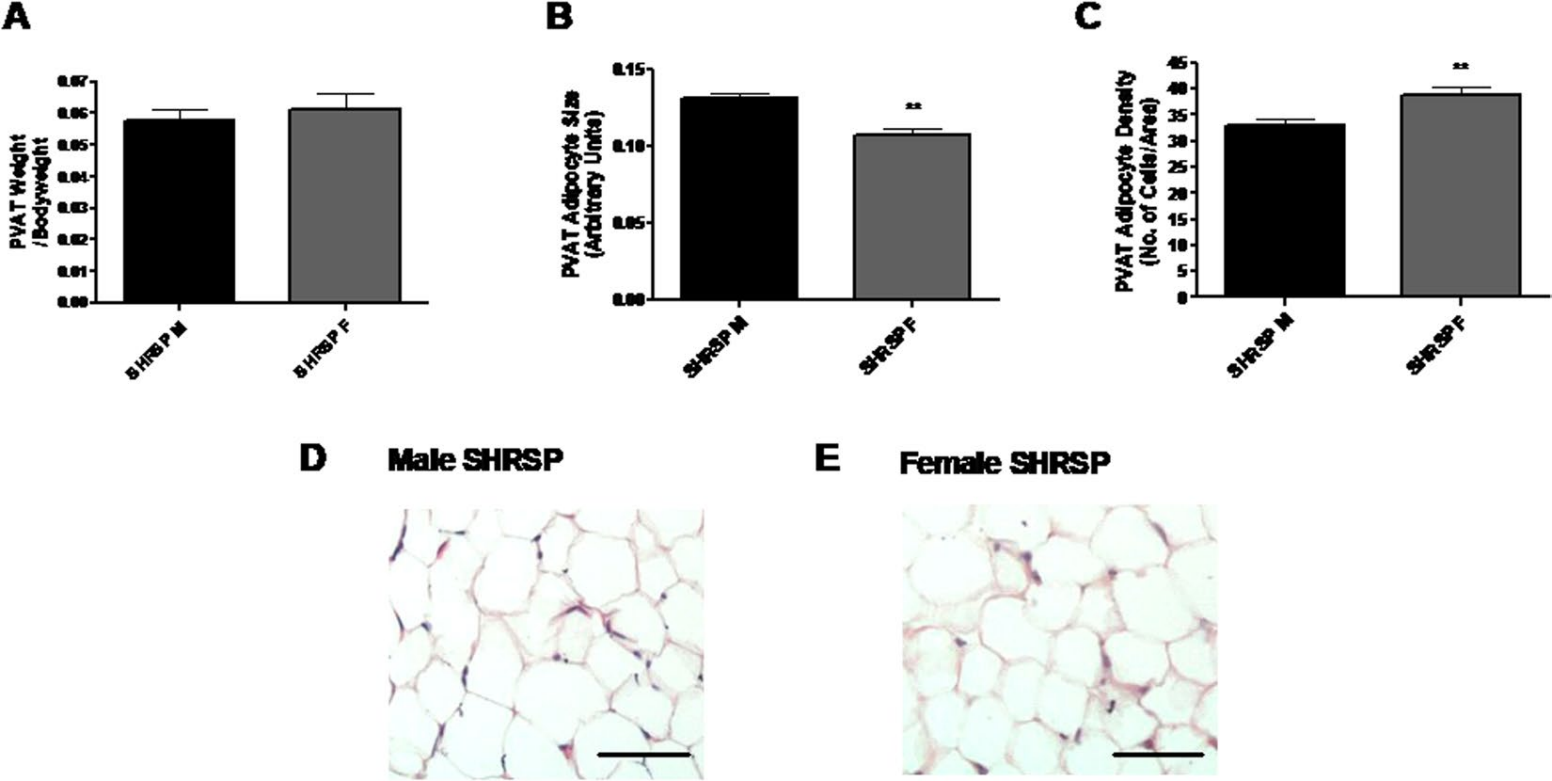
SHRSP males have larger adipocytes in the PVAT surrounding the third-order mesenteric arteries relative to females. PVAT surrounding the mesenteric artery was weighed (3 vessels/animal) in age matched (16 weeks old) male (n = 10) and female (n = 10) SHRSP. When normalised to body weight there was no significant difference in the amount of PVAT surrounding the mesenteric arteries between male and female SHRSP (**A**). On histological analysis, adipocytes size was greater and adipocyte density lesser in the male SHRSP relative to the female SHRSP (**B**,**C**) (**p < 0.01; Student’s t-test). Representative histology sections of the PVAT adipocytes are shown in (**D**,**E**). Scale bar represents 50 μm.

There was no difference in the weight of PVAT which surrounded the mesenteric arteries between male and female SHRSP (Fig. 2A). Histological analysis using haematoxylin and eosin stain revealed that male SHRSP adipocytes were larger in size and reduced in density relative to females (quantification: Fig. 2B,C; representative images Fig. 2D,E).

### PVAT from SHRSP males has a lesser anti-contractile effect and impairs cromakalim-mediated vasorelaxation in female vessels

**Figure 3:**
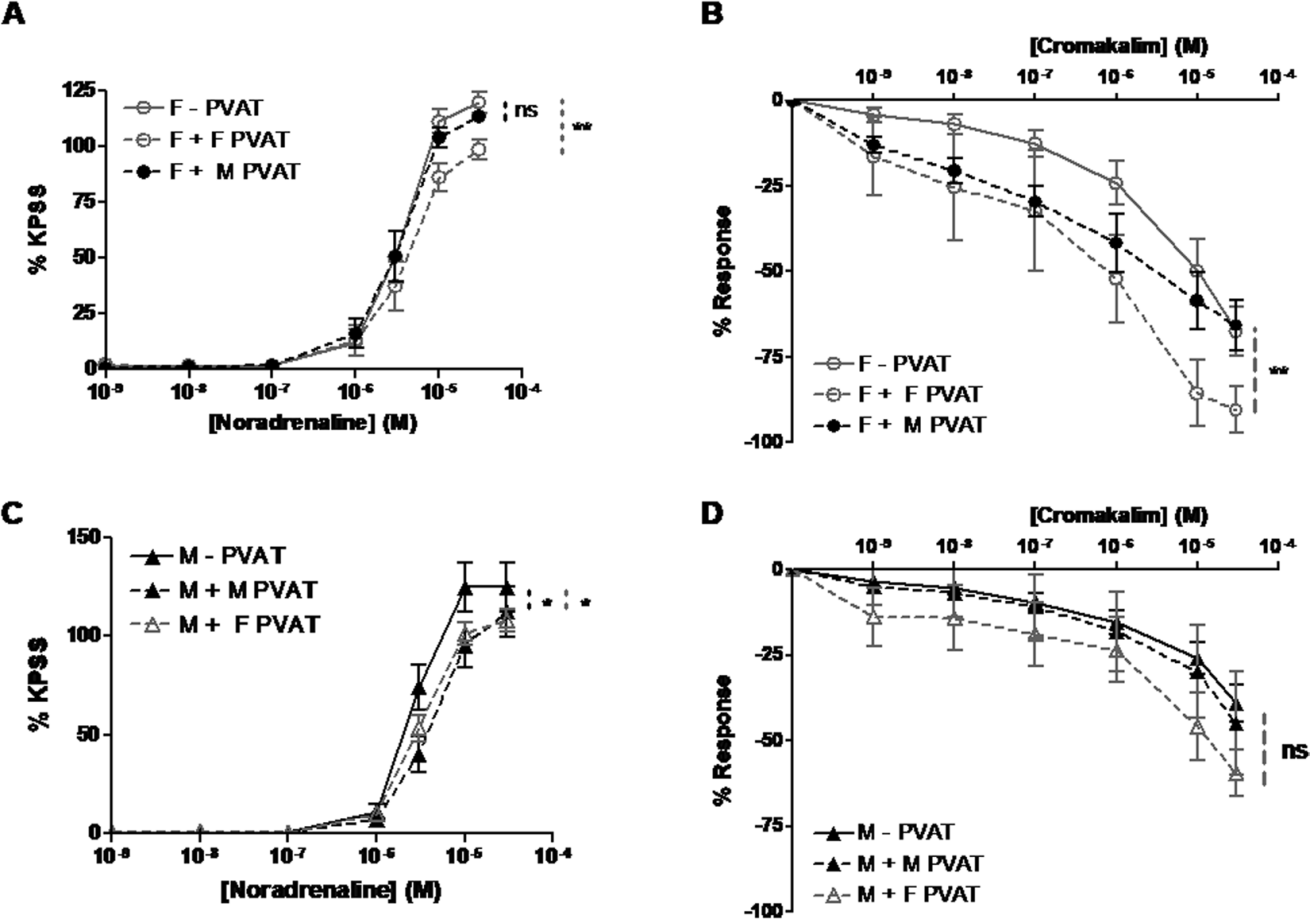
Cross-over of PVAT from male SHRSP alters female third-order mesenteric artery function but not vice versa. Wire myography was used to assess mesenteric artery function in age-matched (16 weeks old) male (n = 10) and female (n = 10) SHRSP where PVAT from mesenteric arteries of SHRSP males was “crossed-over” on to mesenteric arteries (**A**,**B**) of female SHRSP and vice versa (**C**,**D**). Female PVAT had a significant anti-contractile effect on female vessels (**p < 0.01; area under the curve followed by one way ANOVA and post-hoc Tukey test) however this was abolished when male PVAT was used (**A**). Cross-over of male PVAT on to female vessels impaired the vasorelaxation response to cromakalim (**p < 0.01; area under the curve followed by one way ANOVA and post-hoc Tukey test) (**B**). Cross-over of both male and female PVAT had a significant anti-contractile effect on male vessels (*p < 0.05; area under the curve followed by one way ANOVA and post-hoc Tukey test). Cross-over of female PVAT on to the male vessel showed a trend to improve cromakalim-mediated vasorelaxation, however, this was not significant (**D**).

PVAT taken from male SHRSP mesenteric arteries was “crossed-over” to female vessels - PVAT and vice versa. As a control, the PVAT for the same sex vessel was removed and replaced in an identical way. This cross-over method was compared to the endogenous PVAT response which showed it did not alter the response of the vessel (Fig. S4). Cross-over of female PVAT on to a female vessel elicited a significant anti-contractile response, however the reciprocal male PVAT cross-over did not (Fig. 3A). Furthermore, cross-over of male PVAT on to a female vessel impaired the vasorelaxation response to cromakalim (Fig. 3B). Both the cross-over of male and female PVAT on to male vessels had a significant anti-contractile effect (Fig. 3C). The cross-over of male or female PVAT on to male vessels did not have a significant effect on the cromakalim-mediated vasorelaxation; however there was a minor trend for female PVAT to improve vasorelaxation to cromakalim (Fig. 3D).

### Resistin is differentially expressed in PVAT between male and female SHRSP

**Figure 4:**
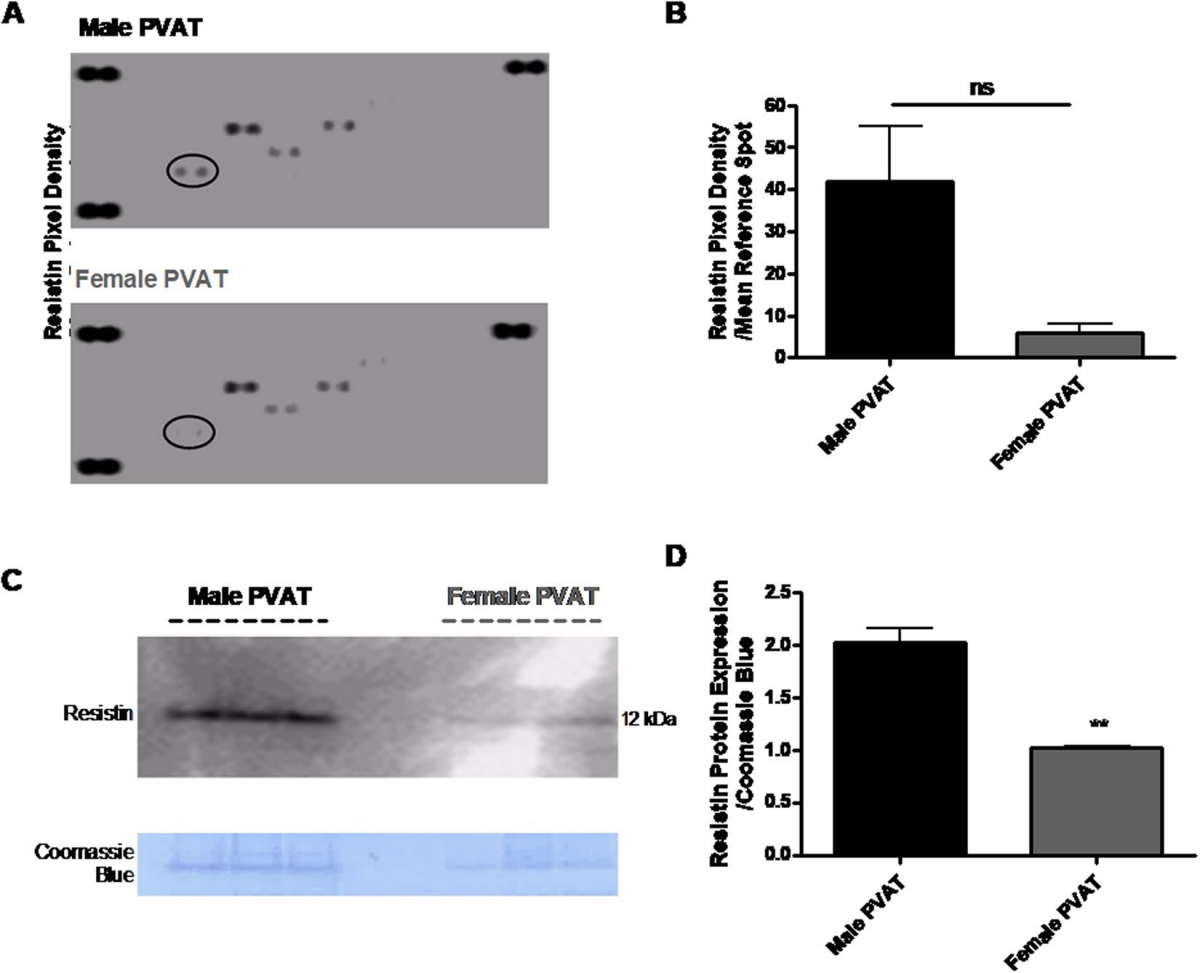
Resistin is increased in PVAT from third-order mesenteric arteries of male relative to female SHRSP. PVAT (perivascular adipose tissue) protein extracts from mesenteric arteries of age-matched (16 week old) male SHRSP (n = 4) and female SHRSP (n = 4) were utilised for an adipokine array (**A**). Each visible spot on the array (**A**) is a technical duplicate from a panel of adipokines which was detected in the PVAT sample. Full results for the adipokine array are shown in Fig. S5. Resistin (identified by a black circle in (**A**)) was identified to be down-regulated in PVAT from female SHRSP relative to male SHRSP (**A**) and quantified in (**B**) (ns; Student’s t-test).

PVAT harvested from third-order mesenteric arteries of age-matched (16 week old) male and female SHRSP was subject to an adipokine array (full results in Fig. S5). The adipokine, resistin, was identified from this screen to show greater expression in PVAT from the male relative to the female SHRSP (Fig. 4A,B).

### Resistin is vasoactive in the mesenteric arteries through impairing K_ATP_ channel mediated vasorelaxation

**Figure 5:**
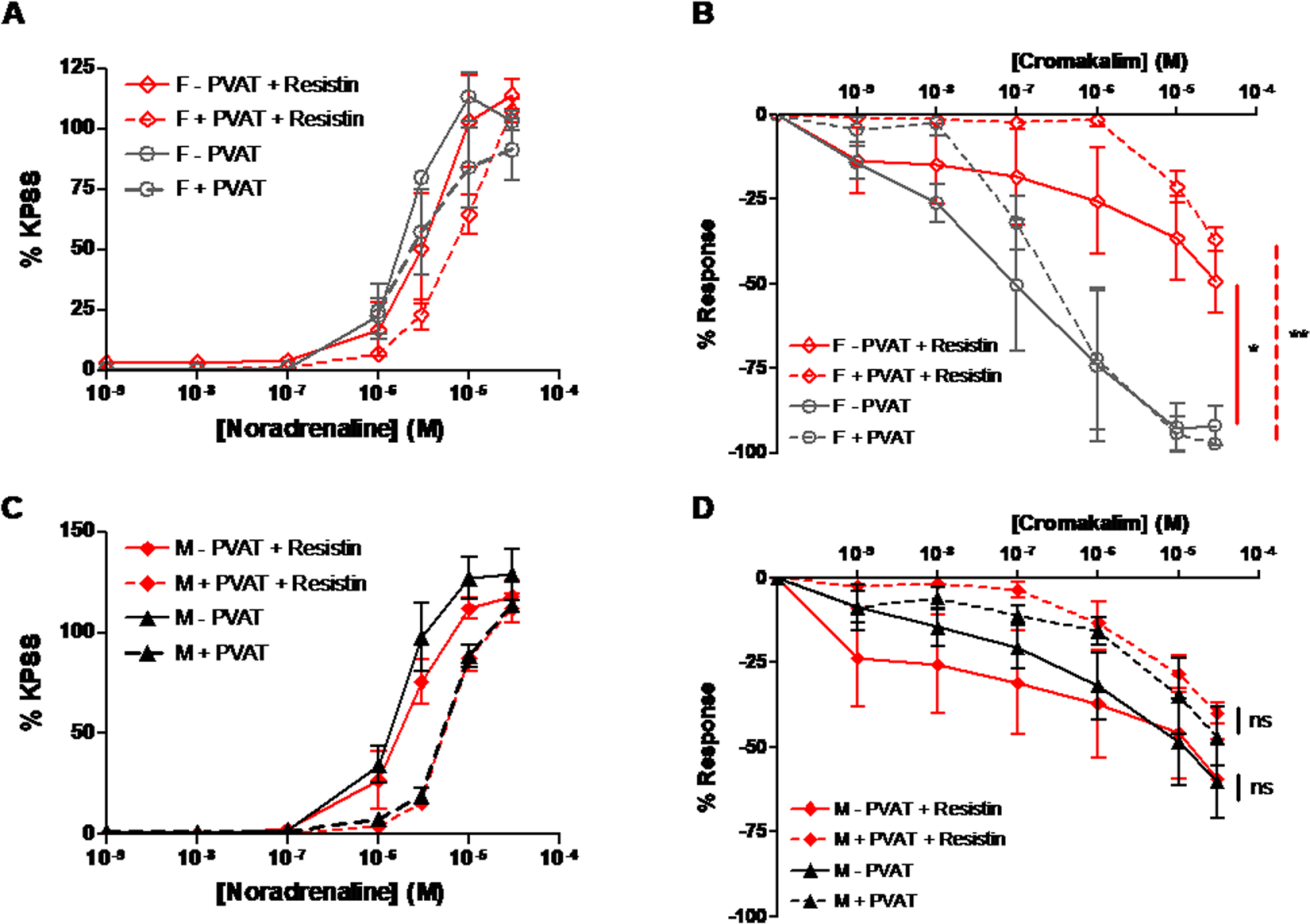
Pre-incubation with resistin inhibits K_ATP_ channel mediated vasorelaxation to cromakalim in third-order mesenteric arteries from female but not male SHRSP. Wire myography was used to assess mesenteric artery function in age-matched (16 weeks old) male (n = 5) and female (n = 5) SHRSP where vessels were pre-incubated prior to drug curves with 40 ng/ml resistin for 4 hours at 37 °C. Pre-incubation with resistin had no significant effect on the contractile response to noradrenaline of the female vessels +/− PVAT (**A**), however, vasorelaxation mediated by cromakalim was significantly impaired in female vessels both+/− PVAT (*p < 0.05, **p < 0.01; area under the curve followed by one way ANOVA and post-hoc Tukey test) (**B**). Pre-incubation with resistin had no significant effects on either the contractile response or the vasorelaxation response to cromakalim in male vessels (ns; area under the curve followed by one way ANOVA and post-hoc Tukey test) (**C**,**D**).

Third-order mesenteric arteries from age-matched (16 week old) male and female SHRSP were pre-incubated with a physiological concentration of recombinant rat resistin (40 ng/ml) at 37 °C for 4 hours before wire myography was used to assess vascular function. Pre-incubation with resistin did not significantly alter the contractile response of female vessels+/− PVAT (Fig. 5A), however, the vasorelaxation response to cromakalim was significantly impaired in both female vessels +/− PVAT (Fig. 5B). In male vessels, resistin pre-incubation did not alter the contractile response to noradrenaline or vasorelaxation response to carbachol (Fig. 5C,D).

## Discussion

Altered PVAT function contributes to the sex-dependent differences in resistance vessel function in the SHRSP through exhibiting a reduced anti-contractile effect in males compared to females. Further, K_ATP_ channel-mediated relaxation was reduced in male third-order mesenteric arteries. Resistin was identified to be up-regulated in PVAT from male vessels compared to females. Incubating female vessels with male PVAT or resistin abrogated K_ATP_ channel-mediated vasorelaxation. These findings establish a novel role for resistin in the sex-dependent effects of PVAT in the setting of hypertension through a K_ATP_ channel-mediated mechanism.

This is the first account in the SHRSP demonstrating reduced anti-contractile effects of PVAT in response to noradrenaline. In agreement with the data presented here, several other models of hypertension such as the spontaneously hypertensive rat (SHR) have been shown to exhibit dysfunctional PVAT^13,15,20^. Overall, the SHRSP have more adipose tissue than WKY and can also present with aspects of metabolic syndrome in contrast with the SHR which is purely a model of hypertension^21^. Increased adiposity has also been associated with reduced vascular function and increased cardiovascular disease which could be construed as a confounding factor in this study. A recent study by Bussey *et al*. showed that maintained weight loss was able to restore PVAT function; i.e. the reduction of PVAT led to the improvement of PVAT function^22^. Adipocytes in PVAT from female SHRSP vessels were significantly smaller than males. Differences in adipocyte size may contribute to a pathogenic function of PVAT since larger adipocytes are limited in further fat uptake and may lead to ‘overspill’ and ectopic fat storage causing formation of reactive oxygen species, endoplasmic reticulum stress and inflammation^23^; all of which have been associated with hypertension^24–26^. A greater number of smaller adipocytes on the other hand provides more storage for lipids^27^. In concordance with the findings, a study that examined abdominal fat of obese women showed an increase in adipose tissue mass was caused by hyperplasia rather than hypertrophy^28^. In this study, there was no difference between SHRSP and WKY with respect to endothelium-dependent vasorelaxation. This was unexpected given that endothelium-dependent vasorelaxation has been shown to be impaired in hypertension.

This study has demonstrated a clear sex-dependent difference between PVAT in male and female SHRSP, which contributes directly to vascular function. PVAT from resistance vessels of female SHRSP retain more anti-contractile ability than males. To dissect whether the apparent reduced anti-contractile effects in females was due to a less dysfunctional female PVAT or improved vessel function compared to males, the cross-over study was devised. These experiments showed that although female vessels have improved vessel function relative to males; female PVAT retains more beneficial effects compared to male PVAT. In contrast, male vessels when incubated with male or female PVAT displayed a similar anti-contractile effect. K_ATP_ channels are important contributors to the regulation of vascular tone^29^; mice lacking K_ATP_ channel subunits display increased hypercontractility and often die from sudden coronary artery spasms^30^. The K_ATP_ channel agonist cromakalim did not result in any difference in relaxation between the WKY and SHRSP males or between WKY males and females. When comparing male and female SHRSP, females were able to relax significantly more regarding both the EC_50_ and maximum relaxation. This suggests a higher expression or preserved function of K_ATP_ channels in female vessels. This could also partially explain the decreased sensitivity of female vessels to noradrenaline stimulation. Corroborating our findings, other hypertensive models have shown a reduced expression of K_ATP_ channel subunits^31^. One limitation of this study is that it was not extended to include a specific K_ATP_ channel such as glibenclamide to provide additional evidence of a K_ATP_ mediated mechanism.

In agreement with our hypothesis that PVAT may play a significant role in the sex-dependent difference in the development of hypertension, a recent study showed that sex can also alter the immune response to exacerbate hypertension. Interestingly, an increased infiltrate of pro-inflammatory T-cells in PVAT was observed only in males in the hypertensive angiotensin II infusion mouse model^32^. The adipokine array identified resistin as a potential candidate for PVAT mediated sex specific effects in vascular function as this was elevated in males compared to females. Resistin, also known as adipose-tissue specific secretory factor (ADSF), is expressed at significant levels in adipocytes, macrophages and mononuclear leukocytes. Its name is derived from its ability to interfere with the action of insulin and is thought to be an important link between obesity and diabetes^33,34^. It has also been linked to other cardiovascular diseases such as atherosclerosis. In addition, it is thought to be involved in the pathology of autoimmune disease, such as rheumatic disease and inflammatory bowel syndrome^35,36^. There has been some debate concerning the translation of these findings discovered predominantly in preclinical models. However, recent work has shown that circulating resistin levels are elevated in both obese individuals with hypertension^37^, and in a seemingly healthy pre-hypertensive population^38^. Further, a recent study suggests resistin contribute to hypertension via TLR4 signalling. Resistin mediated TLR4 signalling can then lead to an NFκB mediated inflammatory response and upregulation of angiotensinogen exacerbating hypertension^39^. The main source of resistin in humans is macrophages rather than adipocytes, but it is well established that macrophages make up a significant portion of the cells found in PVAT^40,41^. Additionally, in inflamed PVAT, macrophages are at least in part responsible for PVAT dysfunction^42^.

The sex -dependent expression of resistin may be affected by the presence of estrogen. Estrogen has been shown to downregulate resistin expression both *in vivo* and *in vitro*^43^ and ovariectomized rats have increased levels of resistin in adipose tissue and serum^44,45^. Contrastingly, testosterone did not affect pituitary resistin levels in mice, suggesting it is the lack of estrogen and not the greater level of testosterone in males that affects resistin expression^46^. However, this relationship has never been explored in the setting of vascular disease. In this study, male and female vessels were pre-incubated with resistin to investigate its direct effect on vascular function. The reported plasma concentration of resistin varies in humans from as low as 4 ng/ml^47^ to 39.9 ng/ml^48^. The relevant concentration of 40 ng/ml was chosen, owing to the relatively short incubation time of four hours needed to ensure viability of the vessels. Resistin did not have any effect on noradrenaline induced vasoconstriction in males or females and did not alter K_ATP_ channel-mediated relaxation in males. However, in females K_ATP_ channel-mediated relaxation was significantly reduced. In line with this result, male PVAT also inhibited K_ATP_ channel mediated relaxation in female vessels in the ‘cross-over’ experiment. This suggests a direct PVAT mediated effect via resistin, which results in reduced K_ATP_ channel mediated relaxation of mesenteric vessels.

The results presented in this study suggest a key role for PVAT in the sex difference in resistance vessel function in the SHRSP which may have a direct role in the development of hypertension in this model. Resistin was identified as a potential mediator of these effects through a novel K_ATP_ channel-mediated mechanism. The significance of resistin in vascular pathophysiology is underpinned by several studies in humans^49–51^. These findings warrant further studies into the molecular mechanism of estrogen’s regulation of resistin expression and how this affects K_ATP_ channel-mediated vasorelaxation. Utilising a sex-specific approach such as this may open up a unique therapeutic avenue for hypertension.

## Supplementary information

Supplementary results

## Data Availability

The authors are happy to make materials, data and associated protocols promptly available to readers.
