# Supplementary material for: Resistin Mediates Sex-Dependent Effects of Perivascular Adipose Tissue on Vascular Function in the Shrsp
Source: Sci Rep. 2019 May 3;9:6897. doi: 10.1038/s41598-019-43326-z (PMC6499830; doi:10.1038/s41598-019-43326-z)
Supplement: Supplementary file 1 — Supplementary results [file 41598_2019_43326_MOESM1_ESM.docx]

**SUPPLEMENTAL MATERIAL FOR:**

**Resistin mediates sex-dependent effects of perivascular adipose tissue on vascular function in the SHRSP**

Sarah McNeilly^1*^, Heather Yvonne Small^1*^, Sheon Mary^1^, Adam Sheikh^1^, Christian Delles^1^

^1^BHF Glasgow Cardiovascular Research Centre, Institute of Cardiovascular and Medical Sciences, University of Glasgow, Scotland

*SMc and HYS contributed equally to this work

**Supplemental Results**

**Supplementary Figure S1: Male WKY and SHRSP have altered mesenteric artery function in the presence of PVAT.** Wire myography was used to assess mesenteric artery function in male 16 week old WKY (n=5) and SHRSP (n=5). PVAT had an anti-contractile effect in both WKY and SHRSP (A). There was a trend for SHRSP vessels – PVAT to be more responsive to noradrenaline than WKY (A). WKY + PVAT vessels were significantly more responsive to contractile stimuli than SHRSP + PVAT vessels (** p<0.01; EC_50_ WKY 4.04 ± 0.74 μM vs. SHRSP 6.13 ± 0.82 μM followed by one way ANOVA and post-hoc Tukey test). With respect to maximum contraction, SHRSP + PVAT vessels had a greater response to noradrenaline relative to the WKY (## p<0.01; one way ANOVA followed by post-hoc Tukey’s test) (B). Furthermore PVAT only had a significant anti-contractile effect with respect to maximum contraction in WKY and not in SHRSP (** p>0.01; one way ANOVA followed by post-hoc Tukey’s test). There was no significant difference between strains or +/- PVAT in vasorelaxation to cromakalim (C). Endothelium-dependent vasorelaxation to carbachol was greater in SHRSP both –/+ PVAT relative to the WKY vessels +/- PVAT (D) (** p<0.01; area under the curve followed by one way ANOVA and post-hoc Tukey test); PVAT did not appear to significantly alter carbachol response in either strain (D). Similarly, vasorelaxation to sodium nitroprusside (SNP) was greater in the SHRSP +/- PVAT vessels relative to the WKY +/- PVAT vessels (E) (** p<0.01; area under the curve followed by one way ANOVA and post-hoc Tukey test); PVAT did not significantly alter the response to SNP in either strain (E).

**Supplementary Figure S2: Male SHRSP have significantly more PVAT surrounding the mesenteric arteries than the WKY.** PVAT surrounding the mesenteric artery was weighed (3 vessels/animal) in male 16 week old WKY (n=5) and SHRSP (n=5). The mesenteric artery of the SHRSP was surrounded by more PVAT when normalised to body weight (A) (*** p<0.005; student’s t-test). On histological analysis, there was no difference in adipocyte size or density between the strains (B-C). Representative histology sections of the PVAT adipocytes are shown in D-E.

**Supplementary Figure S3: Male and female WKY do not exhibit different mesenteric artery function in the absence and presence of PVAT**. Wire myography was used to assess mesenteric artery function in age-matched (16 weeks old) male (n=5) and female (n=4) WKY. Vessels from male and female WKY responded similarly to noradrenaline (A); PVAT reduced the response to noradrenaline in both males and females (A). With respect to maximum relaxation only, vasorelaxation to cromakalim was increased in female + PVAT vessels relative to - PVAT vessels; there was no significant effect in males of PVAT (B). There was no difference in endothelium-dependent vasorelaxation to carbachol in male or female WKY vessels (C). Vasorelaxation to sodium nitroprusside (SNP) was not significantly different between males or females; PVAT did not significantly alter the response to SNP in either strain (D).

**Supplementary Figure S4: The vascular response of the mesenteric arteries is not altered between the endogenous or “crossover” PVAT.** Wire myography was used to assess mesenteric artery function in age-matched (16 weeks old) male (n=10) and female (n=10) SHRSP. Noradrenaline response was not significantly different between vessels which had their endogenous PVAT left on or had “crossover” PVAT i.e. the PVAT removed and then replaced using the crossover method.

**Supplementary Figure S5: Adipokine array in PVAT from the mesenteric arteries of male and female SHRSP.** PVAT (perivascular adipose tissue) protein extracts from mesenteric arteries of age-matched (16 week old) male SHRSP (n=4) and female SHRSP (n=4) were utilised for an adipokine array; 5 adipokines were identified in total (A). Resistin was identified to be down-regulated in PVAT from female SHRSP relative to male SHRSP (B) (ns; Student’s t-test). Insulin-like-growth factor-binding protein 3 (IGFBP3), IGFBP6, lipocalin-2 and intracellular adhesion molecule 1 (ICAM-1) did not show a significant difference between males and females (ns; Student’s t-test).
